# An aberrant species of *Nipponocercyon* from Sichuan, China (Coleoptera, Hydrophilidae, Sphaeridiinae)

**DOI:** 10.3897/zookeys.214.3437

**Published:** 2012-08-06

**Authors:** Martin Fikáček, Sergey Ryndevich, Fenglong Jia

**Affiliations:** 1 Department of Entomology, National Museum, Kunratice 1, CZ-148 00 Praha 4, Czech Republic; 2 Department of Zoology, Faculty of Sciences, Charles University in Prague, Viničná 7, CZ-128 44 Praha 2, Czech Republic; 3 Baranovichi State University, Voykova ul. 21, Baranovichi 225404, Brest obl., Belarus; 4 Institute of Entomology, Life Science School, Sun Yat-sen University, Guangzhou, 510275, Guangdong, China

**Keywords:** Hydrophilidae, Sphaeridiinae, *Nipponocercyon*, taxonomy, morphology, China, Oriental region, Palaearctic region

## Abstract

A detailed examination of specimens of *Cryptopleurum sichuanicum* Ryndevich, 2005 from high altitudes of Sichuan Province, China, revealed that the species belongs in the genus *Nipponocercyon* Satô, 1963 previously endemic to Japan. The species is here transferred in *Nipponocercyon*, and *Nipponocercyon sichuanicus* (Ryndevich, 2005), **comb. n.** is redescribed and compared with *Nipponocercyon shibatai* Satô, 1963. The male genitalia of *Nipponocercyon sichuanicus* is described for the first time. An adapted diagnosis of *Nipponocercyon* is provided, and reasons for the inclusion of *Nipponocercyon sichuanicus* into *Nipponocercyon* and the general distribution of the genus are discussed.

## Introduction

*Cryptopleurum sichuanicum* Ryndevich, 2005 was described from a few female specimens collected in the mountains of Sichuan Province, China (Ryndevich 2005). Although considered “rather peculiar and isolated with some characters differing from other members of the genus”, it was assigned to the genus *Cryptopleurum* based on the combination of large antennal grooves, sculptured dorsal body surface and pentagonal mesoventral plate. Detailed examination of additional material collected more recently revealed that the species shares many characters with the Japanese endemic genus *Nipponocercyon* Satô, 1963 and the superficial similarity with *Cryptopleurum* is due to several unusual apomorphies of the species. *Nipponocercyon* was so far only known from Kyushu, Shikoku and the southern part of the Honshu, where it is represented by a single species, *Nipponocercyon shibatai* Satô, 1963 (Satô 1963, Hoshina and Fikáček 2010). The morphology of the latter Chinese species is compared with *Nipponocercyon shibatai* as well as with the representatives of the genus *Cryptopleurum*, and the reasons for its transfering to *Nipponocercyon* are summarized. The distribution of *Nipponocercyon* is hence extended to the Asian mainland.

## Material and methods

Material examined for this study is deposited in the following collections:

CSR coll. Sergey Ryndevich, Baranovichi, Belarus;

KSEM Natural History Museum, University of Kansas, Lawrence, USA (A. Short);

NHMW Naturhistorisches Museum, Wien, Austria (M. A. Jäch, A. Komarek);

NMPC Department of Entomology, National Museum, Praha, Czech Republic (M. Fikáček);

SYSU Entomological collection of Sun Yat-sen University, Guangzhou, China (F.-L.Jia).

The current study is largely based on newly collected material of *Nipponocercyon sichuanicus* (18 specimens) which were compared with one paratype of *Cryptopleurum sichuanicum*, and on the specimens of *Nipponocercyon shibatai* and of other megasternine genera deposited in the collection of NMPC.

Selected specimens were dissected, with genitalia embeded in a drop of water-soluble dimethyl hydantoin resin on a piece of transparent plastic pinned below the specimen, or of alcohol-soluble Euparal resin on a small piece of glass attached below the respective specimen. The external morphology was examined using the Hitachi S-3700N environmental electron microscope at the Department of Entomology, National Museum in Prague. Habitus photographs were taken using Canon D-550 digital camera with attached Canon MP-E65mm f/2.8 1–5× macro lens, and subsequently adapted in Adobe Photoshop CS2. Figures of genitalia were prepared with the help of Photoshop CS4. The morphological terminology largely follows Komarek (2004) and Fikáček (2010), the higher-level taxonomic nomenclature follows Hansen (1999) and Short and Fikáček (2011).

## Taxonomy

### Nipponocercyon

Satô, 1963

http://species-id.net/wiki/Nipponocercyon

#### Adapted differential diagnosis.

The inclusion of *Cryptopleurum sichuanicum* into *Nipponocercyon* (see below) requires a modification of the differential diagnosis of the genus as follows:

Head without transverse interantennal ridge; eyes small, separated by 9× of one eye; mentum weakly bisinuate on anterior margin; antennae with 9 antennomeres; maxilla with or without sucking disc in males; maxillary palpomere 2 strongly widened distally; posterior tentorial pits minute; pronotum evenly convex, lateral margin not deflexed or slightly deflexed; transverse row of larger punctures along posterior margin of pronotum absent (large areas without microsculpture in *Nipponocercyon sichuanicus* may actually resemble enlarged punctures on the first view, but the punctures are as large as those in disc when examined in detail, see Fig. 8); median portion of prosternum weakly to distinctly separated from lateral portions, bearing coarse setiferous sculpture; median portion of prosternum carinate medially (carina distinct in *Nipponocercyon shibatai*, partly obliterated by the sculpture but still apparent in *Nipponocercyon sichuanicus*, compare Figs 12 and 15); prosternal process wide, deeply excised; antennal grooves moderately large to large, not reaching lateral margin of hypomeron (Figs 11, 14); anteroventral margin of prothorax with a small denticle on the contact of prosternum and hypomeron; profemur with elongate ventral depression along anterior margin; elytron with 10 punctural series; elytral intervals flat or highly convex; lateral margins of elytra not denticulate nor serrate; mesoventral cavities for reception of procoxae large, reaching mesocoxae; preepisternal elevation subpentagonal, widely contacting metaventral process, median portion of metaventrite slightly to very distinctly elevated; postcoxal ridge lying parallel to posterior margin of mesocoxal cavity, not overlapping to lateral margin of metaventrite; lateral portions of metaventrite with coarse punctation (smaller punctures may be intermixed or absent); metanepisternum narrow, but distinct throughout; abdominal ventrite 1 carinate medially, with coarser punctation than ventrites 2–5; phallobase asymmetrical, much shorter than parameres; gonopore situated in basal half of median lobe; male sternite 9 with median tongue-like projection; male sternite 8 without median projection.

A few characters listed as diagnostic for *Nipponocercyon* by Hoshina and Fikáček (2010) have to be excluded as they are only present in *Nipponocercyon shibatai* but absent from *Nipponocercyon sichuanicus*: antennomeres 7–8 with groups of peg-like sensilla ventrally; metaventrite with two short mesal ridges anteriorly (but remnants of ridges seem to be retained in some specimens of *Nipponocercyon sichuanicus*; see the structure indicated as *mtr* in Fig. 20).

#### Recognition.

By the combination of median portion of the prosternum differentiated from lateral portions, subpentagonal preepisternal elevation of the mesothorax widely contacting the metaventrite, large mesothoracic cavities for reception procoxae (reaching to anterior margin of mesoxocal cavity) and metanepisternum well developed both anteriorly and posteriorly, *Nipponocercyon* is most similar to the genus *Australocyon* Hansen, 1990. It may be easily distinguished from the Australian and Neotropical species of *Australocyon* by the male sternite 9 with tongue-like median portion (Fig. 4c), and male sternite 8 without median projection; from *Australocyon pilocnemoides* group it may be distinguished by the undifferentiated surface of the subpentagonal mesoventral plate (with a semicircular median portion defined by a wide bead in *Australocyon pilocnemoides* group, see Fig. 6 in Hansen (2003)), unmodified antennal morphology (in contrast to long antennae with prolonged antennal club pointed at apex and antennomere 6 not cup-like in shape in *Australocyon pilocnemoides* group), and the excavate ventral surface of profemora (without any sculptured depression in *Australocyon pilocnemoides* group).

When the size of mesoventral cavities for reception of procoxae is not taken into consideration, *Nipponocercyon* may resemble other megasternine genera with small subpentagonal mesoventral plate, clearly defined prosternal plate and male sternite 9 tongue-like medially (characters distinguishing the respective genus from *Nipponocercyon* are listed in parentheses: *Agna* (prosternal plate without deeply excised prosternal process, antennal grooves very small and angular in shape, profemur without sculptured depression); *Bolbonotum* and *Kahanga* (elytral grooves deep and wide, reaching total base of elytra, prosternal plate projecting both anteriad and posteriad, profemur without ventral impression, mesoventral plate rhomboid when examined in detail, gonopore apical), *Deltostethus* (mesoventral plate with wide marginal bead, profemur without ventral depression, gonopore apical), and *Pelocyon* (metavetrite with complete femoral lines, prosternal plate longer than wide). *Nipponocercyon sichuanicus* may resemble some species of the genera *Cryptopleurum*, *Pachysternum* and *Cyrtonion* by its large antennal grooves, large grooves for reception of procoxae, reduced epipleura and strongly sculptured body. See below under that species for characters distinguishing it from the mentioned genera.

#### Composition and distribution.

The genus now includes two species, one distributed in Kyushu, Shikoku and the southern part of the Honshu, the other occuring in high altitudes of the mountain ranges in the Chinese province of Sichuan (Fig. 23).

#### Key to *Nipponocercyon* species

**Table:** 

1	Body uniformly brown (Figs 1–2). Elytral intervals strongly convex (Figs 1–2), whole dorsal surface strongly microsculptured (Figs 7–9). Male maxilla without sucking disc. Antennal club without ventral groups of peg-like sensilla (only seen at high magnifications!). Antennal grooves large, nearly reaching lateral margin of hypomeron (Fig. 11). Prosternal plate with obsolete median carina. Preepisternal elevation of mesothorax slightly wider than long (Fig. 19). Anteromedian portion of metavetrite without (or with at most very weakly developed) two short longitudinal ridges, lateral portions with very coarse punctures (Fig. 20). First abdominal ventrite with setiferous punctures many times larger than on ventrites 2–5 (Fig. 21). Protibia angulate distally. Median lobe wide apically (Fig. 4b)	*Nipponocercyon sichuanicus* (Ryndevich, 2005), comb. n.
–	Elytra pale reddish with darker spots at midlength, head and pronotum dark brown (Hoshina and Fikáček 2010, Figs 1–2), dorsal surface with weak mesh-like microsculpture in elytra in some specimens. Elytral intervals flat (Hoshina and Fikáček 2010, Figs 17–18). Male maxilla with sucking disc. Antennal club with ventral groups of peg-like sensilla (only seen at high magnifications!, see Hoshina and Fikáček 2010, Figs 3–4). Antennal grooves moderately large, not reaching close to lateral margin of the hypomeron (Fig. 14). Prosternal plate with very distinct median carina. Preepisternal elevation of mesothorax as wide as long or slightly longer than wide (Hoshina and Fikáček 2010, Figs 11–14). Anteromedian portion of metaventrite with two short longitudinal ridges (Hoshina and Fikáček 2010, Figs 11–14), lateral portions with coarse punctures intermixed with small ones (Hoshina and Fikáček 2010, Fig. 16). First abdominal ventrite with setiferous punctures slightly larger and denser than punctures on ventrites 2–5 (Hoshina and Fikáček 2010, Fig. 19). Protibia rounded distally. Median lobe narrow apically (Fig. 6b)	*Nipponocercyon shibatai* Satô, 1963

### Nipponocercyon sichuanicus

(Ryndevich, 2005) comb. n.

http://species-id.net/wiki/Nipponocercyon_sichuanicus

**Figures 1–2.**
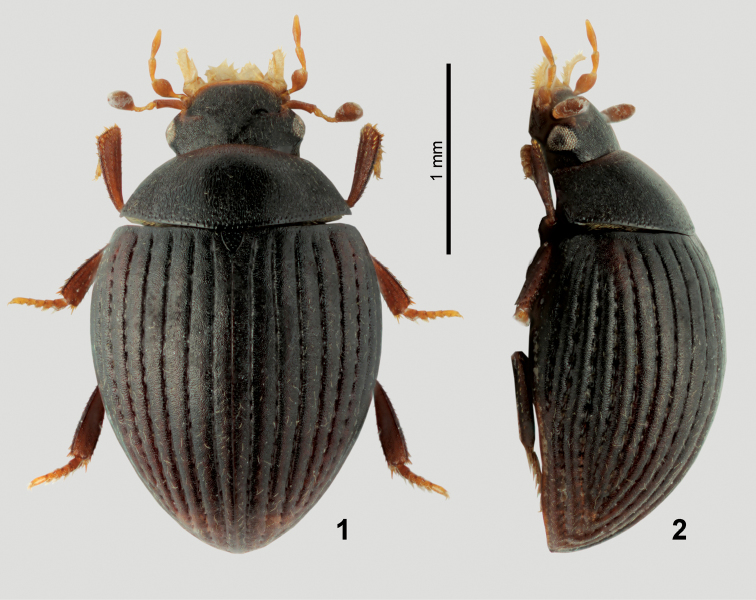
General habitus of *Nipponocercyon sichuanicus*. **1** dorsal view **2** lateral view.

**Figures 3–6.**
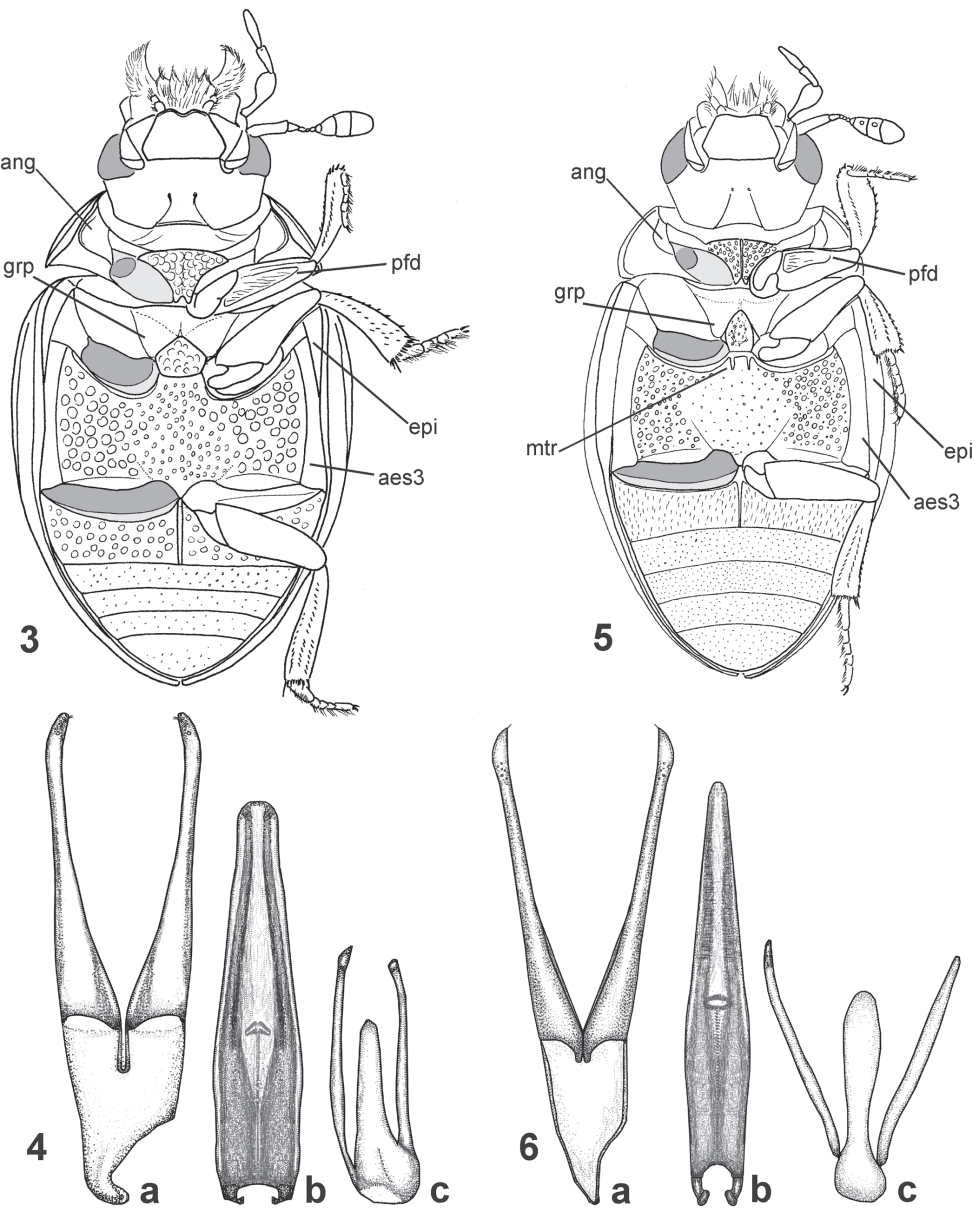
Morphological details of *Nipponocercyon* species. **3–4** *Nipponocercyon sichuanicus*:**3** ventral view **4** male genitalia; **5–6** *Nipponocercyon shibatai*: **5** ventral view **6** male genitalia [the drawing is based on that by Hoshina and Fikáček (2010) in combination with the photo sent to us by H. Hoshina, we did not examine any male specimen for this study]). Parts of male genitalia: **a** tegmen, **b** median lobe, **c** sternite 9. Abbreviations: **aes3** metanepisternum, **ang** antennal groove, **epi** epipleuron, **grp** grooves for reception of procoxae, **mtr** anteromesal ridge of metaventrite, **pfd** profemoral depression.

Figs 1 –4 7–12 19–21 23–24

Cryptopleurum sichuanicum Ryndevich, 2005: 244
Cryptopleurum sichuanicum : Short and Hebauer (2006: 348, catalogue).

#### Type material examined.

Paratype: 1 female (CSR): 'CH, S Sichuan, near / Bijishan Village, left / tr. of Lianhegou River / 2500–3200 m, 19.6.2000 / Belousov, Kabak, Davidian // Paratype / Cryptopleurum / sichuanicum / Ryndevich S. K. // Coll. / SKR // Cryptopleurum / sp.n. / det HEBAUER'.

#### Additional material examined.

**CHINA: Sichuan:** 2 males, 1 spec. (CSR, NMPC): 2.1 km N of Dengsheng, SE of Balanguan Pass, elev. 3455 m, 30°53'3"N, 102°58'23"E, 29.viii.2004, lgt. Belousov & Kabak; 1 spec. (NHMW): 20 km N Sabdȇ, elev. 3300 m, 29°35'N, 102°23'E, 14.vii.1998, lgt. A. Smetana (C82); 1 spec. (CSR): S of Musu village, elev. 2850 m, 31°56'53"N, 103°15'11"E, 19.viii.2007, lgt. Belousov & Kabak; 1 male (NMPC): N Sichuan, SW of Baima, elev. 2980–3040 m [ca. 27°55'N, 101°56'E], 23.vi.2006, lgt. I. Kabak; 5 spec. (CSR, NMPC, SYSU, KSEM): SW of Jiabi, elev. 3240 m, 31°30'40"N, 102°43'43"E, 8–13.viii.2007, lgt. Belousov & Kabak; 1 male, 3 spec. (NHMW, NMPC): Ganzi, Daxue Shan, Mugecuo, ca. 26 km NW Kangding, elev. 3200–3400 m, 30°06'36"N, 101°31'12"E, 21.v.1997, lgt. A. Pütz.

#### Redescription.

Body widely oval, widest in anterior third of elytra. Body length 2.2–2.9 mm, body width 1.4–1.7 mm.

General coloration of dorsal surface dark brown, anterior and anterolateral margins of clypeus and lateral portions of frontoclypeal suture pale reddish, anterior margin of pronotum widely reddish, each elytron slightly paler in humeral area and at elytral apex. Ventral surface dark brown, mentum, mouthparts and posterior portions of temporae reddish brown. Maxillary palpi, antennae and legs pale reddish brown.

Head. Clypeus widely rounded, constricted above antennal bases, with very distinct marginal bead. Dorsal surface of clypeus and frons with strong scale-like microsculpture obscuring the punctation, sparsely arranged punctures not apparent among microsculpture, only evident according to long thin setae arising from punctures. Frontoclypeal suture apparent as a non-sculptured stripe directing mesad, strongly bent posteriad submesally. Mentum with sparsely arranged fine setiferous punctures medially and posteriorly, interstices with strong scale-like microsculpture. Antennal club without distinct groups of peg-like sensilla dorsally or ventrally. Maxilla of male without sucking disc ventrally.

Prothorax. Pronotum with sparsely arranged fine setiferous punctures, larger punctures along posterior margin absent. Whole dorsal surface with mesh-like microsculpture, microsculpture strong along anterior and posterior margins and on lateral portions of pronotum, obsolete on pronotal disc; pronotal disc with irregular longitudinal striae. Lateral portions of pronotum slightly deflexed (and hence seen in ventral view). Prosternum with well defined median plate 2.0× wider than long, bearing strong rugose sculpture, indistinctly carinate mesally. Anterolateral corners of prosternum (at contact with hypomeron) with small but distinct tooth. Antennal grooves large, but not quite reaching lateral margin of hypomeron. Profemur with a rather shallow sculptured depression on a large portion of ventral surface. Protibia angulate distally.

Mesothorax. Scutellar shield with sparse fine punctation, without microsculpture. Elytra with 10 punctural series, all series deeply impressed, lateral striae deeper than median ones; serial punctures minute and rather inconspicuous; elytral intervals highly convex, bearing sparsely arranged fine setiferous punctation, interstices with strong microsculpture consisting of small bumps; lateral portions of elytra deflexed laterally (hence, visible in ventral view); epipleuron present only on elytral base, reduced to extremelly narrow stripe behind level of mesocoxae. Mesoventrite with pentagonal posteromedian elevation, the elevation 1.3× wider than long, with rugose setiferous sculpture.

Metathorax. Anteromedian process with very weakly developed short longitudinal ridges, in many individuals completely obscured by microsculpture; median portion of metaventrite slightly elevated bearing densely arranged coarse setiferous punctures separated by 0.5–1.2× puncture diameter; lateral portions of metaventrite with extremelly large setiferous punctures; whole surface of metaventrite except its posteromedian portion with mesh-like microsculpture on interstices, microsculpture stronger laterally than medially. Hind wings well developed.

Abdomen. All abdominal ventrites with strong scale-like microsculpture, punctation of ventrite 1 consisting of extremelly large setiferous punctures similar to that on lateral portions of metaventrite; punctation of ventrites 2–5 sparse and very fine, nearly completely obscured by microsculpture.

Male genitalia. Parameres slender, 1.8× longer than phallobase. Median lobe robust, very wide and parallel-sided in basal 0.35, slighly and continually narrowing apicad in apical 0.65, apex widely rounded; gonopore situated in basal 0.4 of median lobe. Stenite 9 with slightly asymmetrical median projection.

**Figures 7–18.**
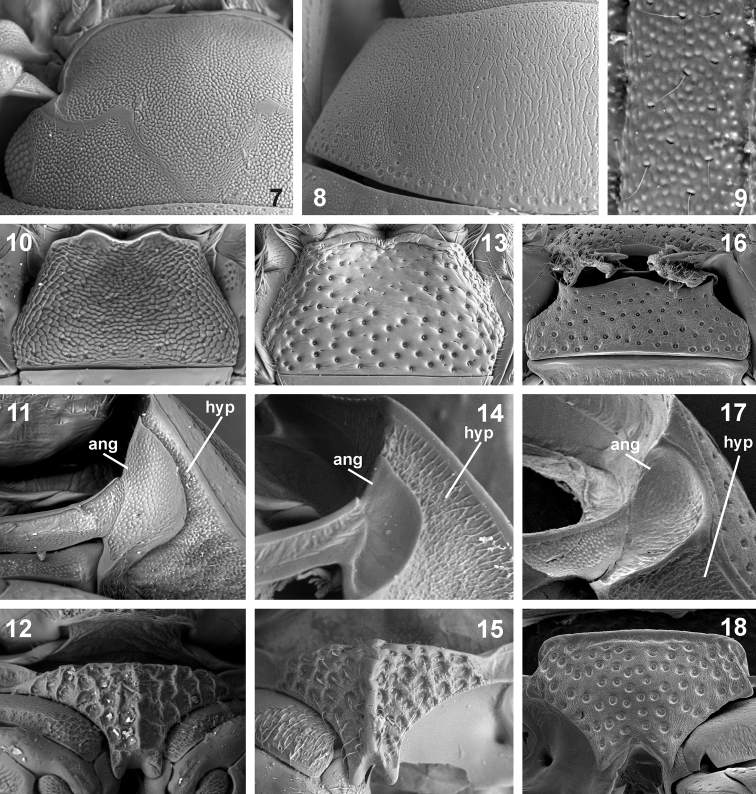
Morphological details of *Nipponocercyon sichuanicus* and its comparison with *Nipponocercyon shibatai* and *Cryptopleurum minutum*. **7–12** *Nipponocercyon sichuanicus*:**7** head in dorsal view **8** pronotum **9 **superficial microsculpture of elytral intervals **10** mentum **11** antennal groove **12** median portion of prosternum. **13–15** *Nipponocercyon shibatai*:**13** mentum **14** antennal groove **15** median portion of prosternum. **16–18** *Cryptopleurum minutum*:**16** mentum **17** antennal groove **18** median portion of prosternum. Abbreviations: **ang** antennal groove, **hyp** hypomeron.

#### Differential diagnosis.

**Figures 19–22.**
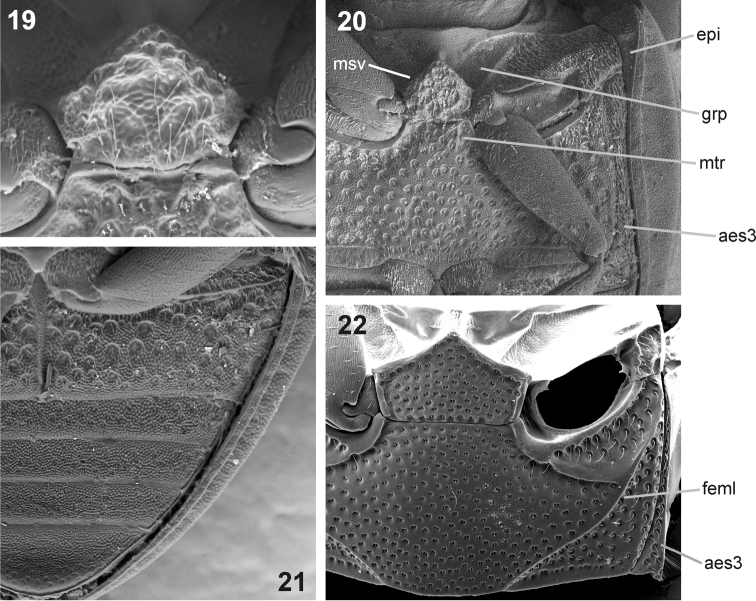
Ventral morphology of *Nipponocercyon sichuanicus* and its comparison with *Cryptopleurum ferrugineum*. **19–21** *Nipponocercyon shibatai*:**19** mesoventral plate **20** meso- and metaventrite **21** abdominal ventrites. **22** meso- and metaventrite of *Cryptopleurum ferrugineum*. Abbreviations: **aes3** metanepisternum, **epi** epipleuron, **feml** femoral line, **grp** groove for reception of procoxae, **msv** mesoventral plate, **mtr** anteromedian ridge of metaventrite.

See the identification key above for characters distinguishing *Nipponocercyon sichuanicus* from *Nipponocercyon shibatai*. *Nipponocercyon sichuanicus* may be confused with some species of *Cryptopleurum*, *Pachysternum* or *Cyrtonion* (the latter not occurring in Asia, however)which are also characterized by large antennal grooves and strongly sculptured dorsal surface. *Nipponocercyon shibatai* may be easily distinguished from them by the combination of following characters: (1) metaventrite without femoral lines (femoral lines present in *Cryptopleurum*, *Pachysternum* and *Cyrtonion*, see e.g. Fig. 22, feml); (2) metanepisternum wide throughout (Fig. 20) (reduced anteriorly and widening posteriad in the above genera as well as in all other genera of the *Megasternum* group characterized by large antennal grooves, see e.g. Fig. 22, aes3); (3) gonopore situated in basal portion of the median lobe (Fig. 4; this character distinguishes both species of *Nipponocercyon* from all other Megasternini); (4) mesoventral plate only slightly wider than long, without acute angles (Fig. 19) (mesoventral plate large and distinctly transverse in *Cryptopleurum*, see e.g. Fig. 22); (5) anterolateral corners of mentum not sharply angulate (Fig. 10) (sharply angulate in *Cryptopleurum*, as in Fig. 16).

#### Biology.

No details on the biology are known. The terrestrial habits of *Nipponocercyon shibatai* (Hoshina & Fikáček 2010) as well as the vast majority of the megasternine taxa suggest that *Nipponocercyon sichuanicus* is a terrestrial species.

#### Distribution.

The species occurs in the mountains of the Sichuan province in South China, at altitudes between 2500–3500 m a.s.l. (Fig. 24).

**Figures 23–24.**
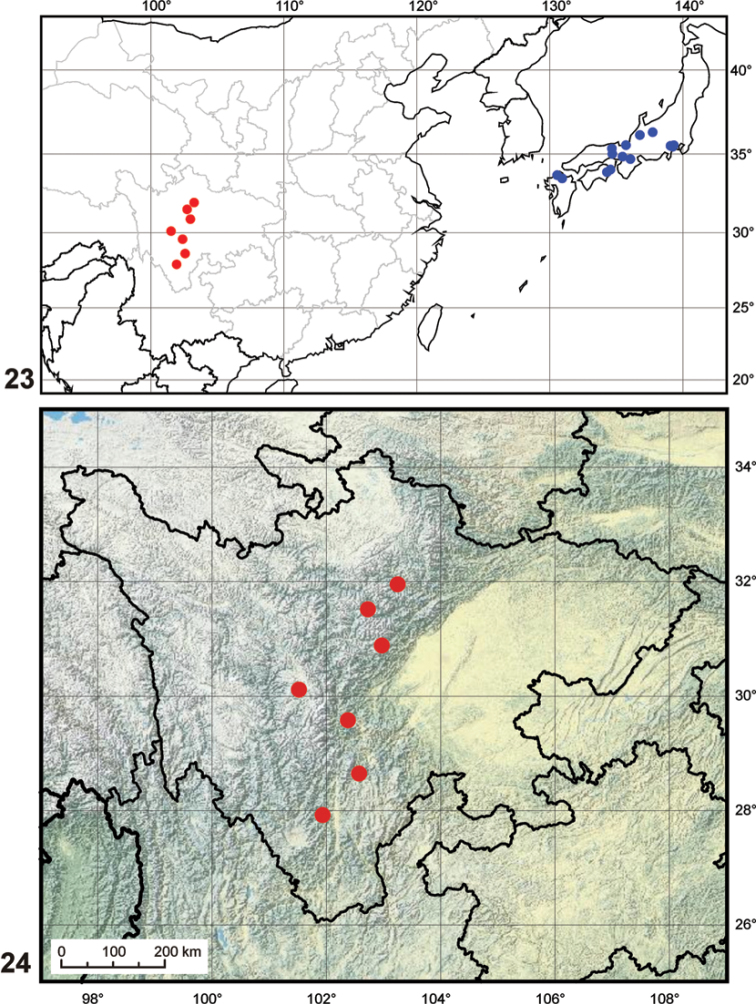
Maps. **23** generaldistribution of the genus *Nipponocercyon* (data for *Nipponocercyon shibatai* adopted from Hoshina and Fikáček 2010) **24** distribution of *Nipponocercyon sichuanicus* in the Sichuan Province. Symbols: **red dot** *Nipponocercyon sichuanicus*, **blue dot** *Nipponocercyon shibatai*.

## Discussion

The inclusion of *Cryptopleurum sichuanicum* into the genus *Nipponocercyon* may appear contradictory, as the species differs in its external morphology from *Nipponocercyon shibatai* in many characters, some of which were previously considered as diagnostic at the generic level. However, a detailed comparison reveals that despite many differences, both species are very similar in terms of the morphology of the ventral side and genitalia (compare Figs 3–6) which is crucial for generic assignment of the megasternine taxa. Especially important in this respect are characters which are present in both *Nipponocercyon* species but absent from all other Megasternini: the basal position of the gonopore on the median lobe (Figs 4b, 6b), and the presence of a depression on the ventral surface of profemur (Figs 3, 5, pfd). Another character infrequent in the Megasternini but shared by both *Nipponocercyon* species is the presence of large mesothoracic grooves for the reception of the procoxae (Fig. 20, grp). Except *Nipponocercyon*, large grooves are only present in *Australocyon* and the *Megasternum* group of genera, of which the representatives of the latter generic group and part of *Australocyon* clearly seem unrelated to *Nipponocercyon* based on other external characters (see the differential diagnosis of *Nipponocercyon* for details). Moreover, general morphology of the genitalia of both speciesis very similar, with principal differences found only in the shape of the apical portion of the median lobe. Eyes with extremelly small dorsal portion are also unusual within the Megasternini but shared by both species. All these characters indicate that both species are more closely related to each other than they are to other megasternine taxa, which justifies the tranfer of *Cryptopleurum sichuanicum* into *Nipponocercyon*.

Characters in which *Nipponocercyon sichuanicus* is contradicting the diagnosis of the genus *Nipponocercyon* used by previous authors (Satô 1963, Hansen 1991, Hoshina and Fikáček 2010) seem now to be autapomorphies of *Nipponocercyon shibatai* or *Nipponocercyon sichuanicus*. For example, the groups of peg-like sensilla on antennomeres 7–8 are autapomorphies of *Nipponocercyon shibatai*; highly sculptured dorsal and ventral surfaces, reduced epipleura (Fig. 20, epi), extremely large antennal grooves (Fig. 11), and the absence of male sucking discs on the maxilla are autapomorphies of *Nipponocercyon sichuanicus*. The reduction of sucking discs in *Nipponocercyon sichuanicus* may be possibly correlated with the strong microsculpture on dorsal surface of this species, which makes the suckers uneffective for male's holding on the female during the copulation. Two diagnostic characters are seemingly absent from *Nipponocercyon sichuanicus*,but a careful examination of multiple specimens revealed that they are present in both *Nipponocercyon* species: remnants of a pair of short anteromesal ridges on the metaventrite (present even in some specimens of *Nipponocercyon sichuanicus*; Fig. 20, mtr) and the median carina of prosternal plate.

The autapomorphies of *Nipponocercyon sichuanicus* make the species similar in habitus to *Cryptopleurum* and related genera, which is the reason why the species was originally described within *Cryptopleurum*. However, a detailed examination shows that this similarity is due to the parallelism, as the body parts which are responsible for the *Cryptopleurum*-like appearance of *Nipponocercyon sichuanicus* differ between *Cryptopleurum* and *Nipponocercyon sichuanicus* in detailed morphology:

(1) The antennal grooves of *Nipponocercyon sichuanicus* are large, but still do not reach the lateral margins of hypomeron, as they do in *Cryptopleurum* and related taxa (compare Figs 11 and 17).

(2) The meso- and metathorax of *Nipponocercyon sichuanicus* resemble *Cryptopleurum* and related genera by their extremely coarse punctation and large grooves for reception of procoxae, but the overall thoracic morphology is totally different from these genera due to its well developed metepisternum (not reduced anteriorly, Fig. 20: aes3) and the ventral side of mesothorax not steeply declined, possessing unmodified mesepimera.

(3) The prosternal plate of *Nipponocercyon sichuanicus* is feebly carinate even though the carina is partly obscured by the strong microsculpture (ecarinate in *Crypopleurum* and related genera, see Fig. 18).

The above differences indicate that despite its superficial resemblance, *Nipponocercyon sichuanicus* is not related to the *Cryptopleurum* group of genera, but probably represents another example of highly sculptured sphaeridiine taxon derived from a non-sculptured ancestor. The shift from non-sculptured to sculptured phenotype was shown to lead to similar ‚sculptured‘ morphology in distantly related taxa in the Megasternini (*Oosternum* and *Emmidolium*, see Fikáček 2007), and similar examples also exist in some other groups of the Sphaeridiinae (e.g., the omicrine genera *Noteropagus* and *Peratogonus* resemble the megasternine genus *Cryptopleurum* by the same characters which are responsible for the *Cryptopleurum*-like appearance of *Nipponocercyon sichuanicus*).

The inclusion of *Cryptopleurum sichuanicum* into *Nipponocercyon* extends the range of the genus (previously endemic to Japan) to the mainland Asia. The isolated occurrence of the genus at high altitudes of the mountain ranges in Sichuan and in mountain areas of southern Japan (Fig. 23) suggests that the current distribution may represent relictual remnants of the former wider distribution of the genus. The situation hence resembles that of the myxophagan genus *Satonius* Endrödy-Younga, 1997 which was originally considered as Japanese endemic (Satô 1963; Endrödy-Younga 1997), but was later found to be distributed in various areas in central and southern China (Hájek and Fikáček 2008; Hájek et al. 2011). High altitude areas of other mountain ranges of China need to be sampled in order to test if the occurrence of *Nipponocercyon* on the continent is really restricted to Sichuan, or if the genus is more widely distributed in the mountains of the transitional zone between Palaearctic and Oriental regions.

Hoshina and Fikáček (2008) examined 35 specimens of Japanese *Nipponocercyon* from the entire Japanese range of the genus, including the types of all three taxa described from Japan by Satô (1963) and Nakane (1968): *Nipponocercyon shibarati* Satô, 1963, *Nipponocercyon shibarati omayanum* Nakane, 1968 and *Nipponocercyon monticola* Nakane, 1968. They found a wide variation in several characters among the specimens they examined (especially the shape of mesoventral plate and metaventral ridges, the presence of dorsal sculpture and the impression of elytral series) which was considered as intraspecific variation due to a weak correlation of the characters and extreme uniformity of male geniatalia. The above taxa were therefore synonymized with *Nipponocercyon shibatai*, although the authors expressed the need for more detailed studies based on more extensive material to clarify the taxonomy of Japanese *Nipponocercyon*. The inclusion of *Nipponocercyon sichuanicus* into the genus may bring new insights into the problem. We have examined specimens of *Nipponocercyon sichuanicus* from a rather wide area in Sichuan (Fig. 24), but all of them are very constant in the proportion of the mesoventral plate. This is in contrast to the high variability of the shape of the mesoventral plate of *Nipponocercyon shibatai* proposed by Hoshina and Fikáček (2008). Moreover, *Nipponocercyon shibatai* and *Nipponocercyon sichuanicus* are very similar in the morphology of the aedeagus (the width of the apex of the median lobe is the only relevant difference, in contrast to many differences in external morphology), indicating that genital morphology is very conservative in *Nipponocercyon*. This suggests that no significant differences in aedeagal morphology should be expected among closely related species, whereas the shape of mesoventral plate may be a good indicator of species limits. This further demostrates the need for additional studies of Japanese *Nipponocercyon*, as the existence of more than one species in Japan cannot be excluded.

## Supplementary Material

XML Treatment for Nipponocercyon

XML Treatment for Nipponocercyon sichuanicus

## References

[B1] Endrödy-Younga S (1997) Active extraction of water-dissolved oxygen and descriptions of new taxa of Toridincolidae (Coleoptera: Myxophaga). Annals of the Transvaal Museum 36 (24): 313-332

[B2] Fikáček M (2010) Hydrophilidae: The genus *Kanala* Balfour-Browne (Coleoptera). In: Jäch MA Balke M (Eds). Water beetles of New Caledonia. Part I.Monographs on Coleoptera 3: 365-394

[B3] Hansen M (1999) Hydrophiloidea (s.str.) (Coleoptera). World Catalogue of Insects 2, 416 pp.

[B4] Hansen M (2003) Discovery of *Australocyon* Hansen and *Pilocnema* Hansen (Coleoptera, Hydrophilidae) outside the Australian region. In: Cuccudoro G Leschen RAB (Eds). Systematics of Coleoptera: Papers celebrating the retirement of Ivan Löbl.Memoirs of Entomology International Associate Publisher: 53-84

[B5] Hájek J Fikáček M (2008) A review of the genus *Satonius* (Coleoptera: Myxophaga: Torridincolidae): taxonomic revision, larval morphology, notes on wing polymorphism, and phylogenetic implications. Acta Entomologica Musei Nationalis Pragae 48: 655-676

[B6] Hájek J Yoshitomi H Fikáček M Hayashi M Jia FL (2011) Two new species of *Satonius* Endrödy-Younga from China and notes on wing polymorphism of *S. kurosawai* Satô (Coleoptera: Myxophaga: Torridincolidae). Zootaxa 3016: 51-62

[B7] Hoshina H Fikáček M (2010) Morphological study and taxonomic revision of the genus *Nipponocercyon* (Coleoptera: Hydrophilidae: Sphaeridiinae). Acta Entomologica Musei Nationalis Pragae 50: 117-130

[B8] Komarek A (2004) Taxonomic revision of *Anacaena* Thomson, 1859. I.Afrotropical species (Coleoptera: Hydrophilidae). Koleopterologische Rundschau 74: 303-349

[B9] Nakane T (1968) New or little-known Coleoptera from Japan and its adjacent regions XXVIII. Fragmenta Coleopterologica 21: 85–86.

[B10] Ryndevich SK (2005) A new species of *Cryptopleurum* Mulsant, 1844 from China (Coleoptera: Hydrophilidae). Zoosystematica Rossica 13: 244.

[B11] Satô M (1963) Description of a new hydrophilid-species from Japan (Coleoptera). Kontyû 31: 267-269

[B12] Short AEZ Hebauer F (2006) World catalogue of the Hydrophiloidea – additions and corrections, 1 (1999–2005). Koleopterologische Rundschau 76: 315-359

[B13] Short AEZ Fikáček M (2011) World catalogue of the Hydrophiloidea (Coleoptera): additions and corrections II (2006–2010). Acta Entomologica Musei Nationalis Pragae 51: 83-122

